# The feasibility of centralized application services for perfusion education programs

**DOI:** 10.1051/ject/2024040

**Published:** 2025-03-07

**Authors:** Blaine Johnson

**Affiliations:** 1 Perfusion Services, UChicago Medicine 5841 S Maryland Ave, Ste E500, MC5040 Chicago IL 60637 USA; 2 Perfusion Education Program, University of Iowa Carver College of Medicine 200 Hawkins Dr, C43-Z GH Iowa City IA 52242 USA

**Keywords:** Perfusion Education Programs, Centralized Application Services, Quality Improvement, Certification, Accreditation

## Abstract

The perfusion profession has recently experienced significant advancements in education and practice. Despite competition from other healthcare professions, the demand for perfusionists continues to grow. This work explores the evolving landscape of perfusion education programs in the United States over the past three decades, highlighting the growing number of accredited programs and rising applicant interest. Additionally, this letter further examines the potential benefits of implementing a centralized application service for perfusion education programs, which could streamline the application process and reduce associated costs for prospective students. By analyzing current trends, including a significant rise in the number of Certified Clinical Perfusionists and enrollment in perfusion education programs, this work underscores the importance of enhancing admission mechanisms to meet the future challenges of the profession. These findings suggest that adopting centralized application services may improve accessibility and efficiency in the application process, ultimately supporting the continued growth of the perfusion profession.

## Discussion

Over the past thirty years, there have been significant changes in medicine, medical economics, and medical education. The field of perfusion is dynamic and continuously evolving, and these changes are reflected in its practice. It has been said that “the strength of the perfusion profession is tied to the success of perfusion education programs” (PEPs) [1]. This work aims to examine whether and how the introduction of centralized application services (CASs) could enhance the application process for PEPs in the United States.

The field of perfusion is experiencing significant changes due to rapid advancements in innovative technologies and techniques, which are creating new opportunities for growth and expansion of perfusion practice. In recent years, there has been a steady increase in the number of PEPs in the United States. Concurrently, the number of students applying to and graduating from these programs has also risen substantially, indicating a promising future for the profession.

Since 1969, a variety of perfusion programs have been established at universities and hospitals [2]. By 1994, there were thirty-five PEPs in the United States [3]. As of 2024, twenty-one PEPs have received accreditation from the Commission on Accreditation of Allied Health Education Programs based on recommendations from the Accreditation Committee for Perfusion Education [4]. Additionally, three other programs are currently pending candidate accreditation.

Currently, there are two entry-level degree types in the perfusion profession: a Post-Baccalaureate Certificate (25% [6/24]) or a Master's Degree (75% [18/24]). In 2023, the American Board of Cardiovascular Perfusion administered 259 Perfusion Basic Science Examinations to first-time examinees and 250 Clinical Applications in Perfusion Examinations to first-time examinees [5]. Additionally, the total number of Certified Clinical Perfusionists has increased by 44.5% from 3,375 in 2000 to 4,878 in 2023.

Finding enough qualified applicants is no longer a challenge, despite competition from other life science and allied health professions [6, 7]. Recent trends indicate a significant increase in applications for PEPs. This surge is likely due to the growing demand for qualified perfusionists in the healthcare field, primarily fueled by an aging population and an increase in cardiac surgical procedures requiring perfusion support. These trends have also raised concerns about a potential shortage of perfusionists, further enhancing the number of applications received [8, 9].

Millions of prospective students apply to colleges annually through a wide variety of admission mechanisms. In some countries, such as Japan and the United States, admissions are decentralized, in the sense that students apply to each college separately [10, 11]. In other countries, such as Germany and Brazil, the application and admission process is centralized, and students are assigned to colleges through clearinghouse systems [10, 11]. The use of a CAS clearinghouse has resulted in profound changes in the post-secondary education market by mitigating several application challenges.

Over the last three decades, over 40,000 educational programs have successfully managed admissions through CAS technology [12]. This system simplifies the application process for students by lowering search costs and providing a central platform with information about majors, institutions, and locations. Various CAS platforms are available, catering to fields such as medicine, law, dental, pharmacy, physical therapy, and more [13]. In fact, one current PEP utilizes a CAS known as the Allied Health Centralized Application Service (AHCAS) [14].

AHCAS simplifies the application process for allied health programs [15]. After prospective applicants select the programs they wish to apply to, they can submit a single application containing all required materials, such as fees, exam scores, transcripts, and other documents. Once AHCAS receives the application and materials, they may undergo a verification process. This process ensures that all necessary components are included and the application is complete, saving time and effort for both applicants and the PEPs.

As of 2024, the average application cost for all PEPs was $66.17, based on a total of $1,522 for twenty-three applications (Table 1). If prospective applicants use CASs like AHCAS to apply the same twenty-three PEPs, the average cost drops significantly to $45.22, totaling $1,040 for all applications. This substantial cost reduction is attributed to the CAS’s pricing structure, which charges $72 for the first program applied to and $44 for each additional program. However, it is essential to note that if prospective applicants apply to only one PEP, their costs would be higher through AHCAS because the savings are only realized when applying to two or more PEPs.

**Table 1:** Comparison of degree type and application cost for Perfusion Education Programs in the United States

Sponsor	City, State	Degree type	Application cost
Baylor Scott & White The Heart Hospital at Plano	Plano, TX	Certificate	$100
Cleveland Clinic Foundation	Cleveland, OH	Certificate	$75
Texas Heart Institute	Houston, TX	Certificate	$150 – Initial, $50 - Reapplicant
University of Iowa	Iowa City, IA	Certificate	$100
University of Texas Health Science Center at Houston	Houston, TX	Certificate	$150
Vanderbilt University Medical Center	Nashville, TN	Certificate	$75
Emory University	Atlanta, GA	Masters	$50
Hofstra University	Hempstead, NY	Masters	$75
Keck School of Medicine of USC	Los Angeles, CA	Masters	$90
Lawrence Technological University	Southfield, MI	Masters	$50
Lipscomb University	Nashville, TN	Masters	$50
Medical University of South Carolina	Charleston, SC	Masters	$100
Midwestern University	Glendale, AZ	Masters	$50
Milwaukee School of Engineering	Milwaukee, WI	Masters	FREE
Northern Kentucky University	Highland Heights, KY	Masters	$40
Quinnipiac University	Hamden, CT	Masters	$45
Rush University	Chicago, IL	Masters	$72
SUNY Upstate Medical University	Syracuse, NY	Masters	$65
Thomas Jefferson University	Philadelphia, PA	Masters	$50
University of Arizona	Tucson, AZ	Masters	$85
University of Nebraska Medical Center	Omaha, NE	Masters	$60
University of Texas Health Science Center at Tyler	Tyler, TX	Masters	PENDING
University of Utah	Salt Lake City, UT	Masters	$65
UPMC Presbyterian Shadyside	Pittsburgh, PA	Masters	FREE

## Conclusion

The evolution of the perfusion profession over the past few decades has been marked by significant advancements in education and practice, primarily driven by technological innovations and an increasing demand for qualified perfusionists. The introduction of CASs offers a promising solution to streamline the application process for perfusion education programs. By simplifying the application procedure and reducing costs, CASs may attract more candidates into the field, addressing the growing need for skilled professionals. As the number of accredited programs and graduates continues to rise, implementing such systems could further strengthen the perfusion profession and ensure that it meets the demands of the evolving healthcare landscape. Embracing these changes will be critical for the ongoing growth and sustainability of perfusion education and practice in the United States.

## Data Availability

The data supporting this study’s findings are available from the corresponding author upon reasonable request.
